# Construction of a prognostic signature associated with liver metastases for prognosis and immune response prediction in colorectal cancer

**DOI:** 10.3389/fonc.2023.1234045

**Published:** 2023-07-26

**Authors:** Chang Liu, Zhihua Lu, Jun Yan, Dong Xue, Xiaoyu He, Wenbo Huang, Qi Sun, Wei Zhao, Fanni Li

**Affiliations:** ^1^ Department of General Surgery, The First Affiliated Hospital of Xi’an Jiaotong University, Xi’an, China; ^2^ Department of General Surgery, Qilu Hospital (Qingdao), Cheeloo College of Medicine, Shandong University, Qingdao, China; ^3^ Center for Gut Microbiome Research, Med-X Institute, The First Affiliated Hospital of Xi’an Jiaotong University, Xi’an, China; ^4^ Department of Talent Highland, The First Affiliated Hospital of Xi’an Jiaotong University, Xi’an, China

**Keywords:** colorectal cancer, liver metastases, prognosis, tumor microenvironment, HAMP

## Abstract

**Background:**

As the most common gastrointestinal malignancy worldwide, liver metastases occur in half colorectal cancer (CRC) patients. Early detection can help treat them early and reduce mortality in patients with colorectal cancer liver metastases (CRLM). Finding useful biomarkers for CRLM is thus essential.

**Methods:**

The TCGA and GEO databases were used to download the expression profiles and clinical data of the patients. Differential analysis screened for genes associated with CRLM, and univariate Cox regression analysis identified genes associated with prognosis. The least absolute shrinkage and selection operator (LASSO) method further preferred genes to construct a prognostic signature. Kaplan-Meier survival curves were used to show patients’ overall survival (OS). Receiver operating characteristic (ROC) curves showed the accuracy of the model. Risk scores and clinical characteristics of patients were included in multivariate Cox regression analysis to identify independent risk factors, and a nomogram was constructed. The proportion of immune cells and infiltration were assessed using the ‘CIBERSORT’ package and the ‘ESTIMATE’ package.

**Results:**

We constructed a signature consisting of seven CRLM-associated genes, and signature-based risk scores have great potential in estimating the prognosis of CRC patients. Moreover, the poor response to immunotherapy in high-risk patients might contribute to the poor prognosis of individuals. Furthermore, we found that overexpression of Hepcidin antimicrobial peptide (HAMP), the only gene highly expressed in CRC and liver metastatic tissues, promoted CRC development and that it was associated with tumor mutation burden (TMB), DNA mismatch repair (MMR) genes, and microsatellite instability (MSI) in various tumors. Finally, we found that in CRC patients, low expression of HAMP also represented a better immunotherapeutic outcome, reflecting the critical role of HAMP in guiding immunotherapy.

**Conclusion:**

We identified a prognostic signature containing 7 CRLM-associated genes, and the signature was specified as an independent predictor and a nomogram containing the risk score was built accordingly. In addition, the derived gene HAMP could help guide the exploration of profitable immunotherapeutic strategies.

## Introduction

Colorectal cancer (CRC) ranks second in cancer-related deaths globally (1). 2020 Global Cancer Statistics says there were almost over 900,000 CRC-related deaths worldwide annually, accounting for 10% of new cancer cases and deaths (2). Patients with regional or distal CRC have a noticeably decreased survival rate while having a better prognosis if they do not have metastases (3). CRC is most likely to metastasize to the liver, accounting for 70% of all metastatic cases (4). Half of CRC patients will develop liver metastasis, and one of the reasons for this is the compromised intestinal vascular barrier (5). Bacterial dissemination to the liver after gut vascular barrier injury promotes the formation of pre-metastatic ecotone and facilitates the recruitment of metastatic cells. Early detection is helpful in early treatment and reducing mortality of colorectal cancer liver metastasis (CRLM) patients because, at the time of the diagnosis of liver metastasis, over one-third of CRC patients have progressed into all liver tissues (6). Surgical removal of affected liver tissue while preserving sufficient hepatic function is the only chance of long-term survival for CRLM patients (7). However, most CRC with extensive metastases or other metastatic diseases is unresectable (8). In addition, recurrence of CRC is not uncommon after surgical resection (9). Advancements in understanding CRC have increased the effectiveness of treatment methods, and finding effective biomarkers for CRLM may assist in early treatment management (10).

Immunotherapy can be used for first-line or follow-up treatment of metastatic CRC (11). Patients who respond well to tumor immunotherapy have a better prognosis (12), and predicting the effect of immunotherapy has an essential role in assessing patient prognosis. The infiltration of immune cells in the tumor microenvironment (TME) and the stromal cell proportion play an essential role in promoting tumor development and immunotherapy (13). Therefore, the status of TME can predict the treatment outcome of patients and has an essential impact on the assessment of prognosis. However, the TME encompasses numerous distinct cell types, each exerting different effects on tumor growth and response to immunotherapy. The cellular composition and function within the TME continuously change throughout tumor progression, and both the tumor and its corresponding TME exhibit a high degree of heterogeneity across patients and tumor sites. These properties may contribute to inaccurate predictions of immunotherapy response (14, 15). Although high microsatellite instability (MSI-H) in colorectal cancer patients is considered a significant predictor of response to immune checkpoint inhibitor therapy, not all MSI-H patients benefit from immunotherapy. Studies have shown that only 40-70% of MSI-H colorectal cancer patients are likely to benefit from immunotherapy (16, 17). Furthermore, the percentage of MSI-H colorectal cancer patients is only about 15% (18). Thus, there is a need to find new predictors to guide immunotherapy.

Hepcidin is encoded by hepcidin antimicrobial peptide (HAMP), which controls iron absorption in the intestine and is released from macrophages to maintain iron homeostasis (19). Increased HAMP expression can lead to iron deficiency (20), which can cause the progression of CRC by failing to meet the iron requirement for immune cell functions (21). Besides, the expression of HAMP is increased in CRC samples compared to the normal samples (22). Moreover, hepcidin-ferroportin signaling promotes tumor cell homing, which is critical in tumor metastasis (23). Whether HAMP expression is associated with CRLM still needs further exploration.

## Materials and methods

### Data collection

Expression profile information and relevant clinical information for a total of 622 patients with colon cancer (COAD) and rectal cancer (READ) were downloaded from TCGA (https://tcga-data.nci.nih.gov/tcga/ ) database. Thirty-two data without complete survival information were removed. Mutation data and expression profiles for pan-cancer were also downloaded *via* the TCGA database. The GSE81582 dataset containing expression profiles and relevant clinical information for 19 CRLM and 23 primary CRC samples was downloaded from GEO (https://www.ncbi.nlm.nih.gov/geo/ ) database.

### Differential expression analysis

Differential expression analysis was performed using the ‘limma’ package to select differentially expressed genes (DEGs) (24). The absolute value of logarithmically converted fold change (|Log2FC|) > 0.6 and utilized a false discovery rate (FDR) filtering level of 0.05.

### Prognostic signature construction

Univariate Cox regression analysis identified genes associated with prognosis. least absolute shrinkage and selection operator (LASSO) analysis was performed using the ‘glmnet’ package (25) in R language to screen the genes further. Moreover, the following formula was used to determine the risk score: $risk\;score=\sum_{i=1}^{7}Coef_{i}*X_{i}$ . The $Coef_{i}$ is the risk factor calculated by the LASSO model for each gene, and the $X_{i}$ is the expression of each gene. The TCGA sample data were randomly divided into training and validation groups at a 1:1 ratio. Using the median risk score value, patients inside this training cohort were split into low- and high-risk groups. The survivor and ‘survminer’ package were used to generate overall survival (OS) Kaplan-Meier curves for high- and low-risk groups. To compare the survival curves, the log-rank test was used. The ‘timeROC’ package (26) developed receiver operating characteristic (ROC) curves to evaluate the predicting efficacy of the signature. Decision Curve Analysis (DCA) was performed using the ‘dca.R’ package.

### Nomogram establishment

To create a nomogram for predicting patient outcomes, we utilized the ‘rms’ package. We fitted a multivariate Cox proportional hazards regression model using the significant prognostic factors identified in our study. Subsequently, we employed the ‘cph’ function to estimate the regression coefficients and hazard ratios for each variable. We then used the ‘nomogram’ function to construct a graphical representation of the Cox model, facilitating an intuitive visualization and interpretation of the results. The performance of the nomogram was assessed using calibration plots, which were generated with the ‘calibrate’ function in the ‘rms’ package.

### TME assessment and immunotherapy prediction

The proportion of 22 immune cell infiltrates in CRC patients was calculated using the ‘CIBERSORT’ package. Stromal score, immune score, and tumor purity of malignant tumors were assessed using the ‘ESTIMATE’ package. The expression of several immune checkpoint levels was compared using the Wilcoxon test (27). The immunophenoscore (IPS) was obtained through The Cancer Immunome Atlas (TCIA) (https://tcia.at/home ) database. The tumor immune dysfunction and exclusion (TIDE) score was calculated using the online website TIDE (http://tide.dfci.harvard.edu/ ). Neoantigen data for tumors were obtained from a previous study (28).

### Drug treatment response

The response of each patient to each drug treatment was predicted by estimating the half-maximal inhibitory concentration (IC50) using the ‘oncoPredict’ package, and associations with 198 drugs were calculated using Spearman rank correlation coefficients.

### Expression of HAMP in multiple cancer types

The expression of HAMP in several cell lines was verified using the cancer cell line encyclopedia (CCLE, broadinstitute.org) database. HAMP expression in pan-cancer was observed using TIMER2.0 (cistrome.org). The MSI score for each tumor was obtained from a previous study (29). The TMB of each tumor was calculated using the ‘tmb’ function of the ‘maftools’ package. The MSI, TMB, and gene expression data of the samples were integrated separately for correlation analysis.

### Functional enrichment analysis

GO and KEGG enrichment analyses were performed on the identified DEGs using the ‘clusterProfiler package (30). The adjusted p-value< 0.05 was used as the criterion to filter significantly enriched GO terms and KEGG pathways. The c2.cp.kegg.v7.0.symbols.gmt genome was obtained through the MSigDB database (http://software.broadinstitute.org/gsea/msigdb ). GSEA analysis with the above data. The p-value< 0.05 was used as filtering criteria to screen significantly activated KEGG pathways.

### CRC tissues and immunohistochemistry

Eight pairs of fresh CRC and adjacent normal tissues were obtained from CRC patients treated with radical surgery at the First Affiliated Hospital of Xi’an Jiaotong University. All subjects signed an informed consent form. In compliance with the Helsinki Declaration, the study was approved by the Medical Ethics Committee of the First Affiliated Hospital of Xi’an Jiaotong University. As previously reported, the immunohistochemical staining procedure was performed using the streptavidin peroxidase-conjugated method (SP-IHC) (31). The hepcidin antibody (cat. no. sc-100277) was purchased from (Santa Cruz Biotechnology, Texas, USA).

### Culture of CRC cell line

RPMI 1640 media (Gibco BRL, Carlsbad, CA, USA) supplemented with 10% FBS (Gibco BRL, Carlsbad, CA, USA) was used to maintain human colon cancer cells (DLD-1) and intestinal epithelial cells (FHCs) (Shanghai Institute of Cell Biology, Chinese Academy of Sciences, China). Cells were cultured at 37°C with 5% CO_2_.

The target gene (HAMP) silenced RNA (si-RNA) was constructed by the Life Technologies (Thermo Fisher Scientific), and we transfected the DLD-1 cells with 100 nM siRNA using lipofectamine 2000 (Invitrogen, CA, USA) according to the manufacturer’s protocol (Invitrogen, CA, USA).

### Quantitative real-time polymerase chain reaction

The TRIzol reagent was used to isolate the total RNA of cells (Invitrogen, Carlsbad, CA, USA). Then, the obtained RNAs were used for cDNA synthesis with the PrimeScript RT Reagent kit (TaKaRa, Osaka, Japan). cDNA was next used for qPCR with SYBR Green fluorescence signal detection assays (TaKaRa, Osaka, Japan) on an IQ5 instrument (Bio-Rad, CA, USA). The forward primer for HAMP is *5’- CTGACCAGTGGCTCTGTTTTC -3’*, and the reverse primer is *5’- GAAGTGGGTGTCTCGCCTC-3’*. Using the 2^−ΔΔCT^ approach, the level of mRNA expression was measured.

### Transwell migration assay

The RPMI 1640 medium supplemented with 10% FBS medium was placed in the bottom compartments of a transwell chamber, and RPMI 1640 serum-free medium containing 1*10^5^ transfected DLD-1 cells was added to the upper compartments. After incubation for 24h at 37°C and 5% CO_2_, the upper chambers were emptied and fixed using methanol. The migrating cells were then stained with 0.1% crystal violet. Then the staining was observed under an inverted phase contrast microscope. The migrated cells were counted in five randomly selected high-power visual fields, and their number was averaged. The experiments were repeated to verify the consistency and reliability of the experimental results. Each experiment was performed three times under the same conditions and using the same methods and materials.

### Statistical analysis

The chi-square test was used to compare the composition ratios of categorical variables between the two groups. The log-rank test completed the comparison of each Kaplan-Meier curve. The correlation was evaluated using the Pearson correlation analysis and Spearman correlation analysis. The Wilcoxon signed-rank test completed the comparison of different groups. The statistical analysis was based on the R (4.0.2) programming language. P value< 0.05 was the filtering criterion.

## Results

### Identification of CRLM-related genes

**Figure 1:**
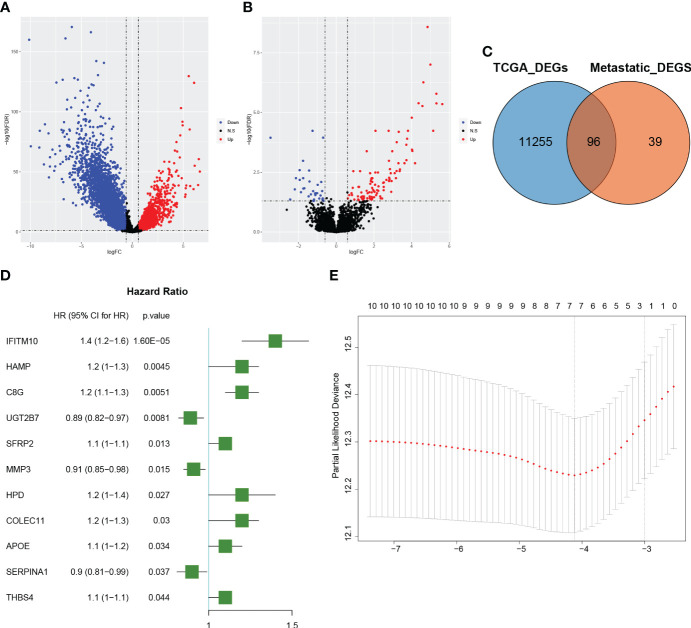
Identification of colorectal cancer liver metastatic (CRLM) related genes **(A)**: The volcano diagram of colorectal cancer (CRC) vs. normal samples in the TCGA dataset. The blue and red dots represent the downregulated and upregulated genes, respectively. **(B)**: The volcano diagram of CRLM samples vs. CRC non-metastatic samples in the GSE81582 dataset. The blue and red dots represent the downregulated and upregulated genes, respectively. **(C)**: Venn diagram depicting the genes associated with CRLM. The blue circle represents differentially expressed genes between CRC and normal samples in the TCGA dataset, while the orange circle represents differentially expressed genes between CRLM samples and non-metastatic CRC samples in the GSE81582 dataset. The overlapping section illustrates the set of genes that are differentially expressed in both datasets. **(D)**: Forest plot illustrating the 11 significant CRLM-associated genes identified through univariate Cox regression analysis. The plot displays each gene’s hazard ratios and corresponding 95% confidence intervals. **(E)**: Partial likelihood deviation plot for LASSO regression. The x-axis shows log(lambda) values, while the y-axis represents partial likelihood deviation values. The dashed line on the left indicates the optimal value of lambda, and the number above each curve corresponds to the number of non-zero coefficients in the model.

There were 1835 up-regulated genes and 9516 down-regulated genes in CRC samples compared to normal tissue in the TCGA dataset (**Figure 1A**), and 102 up-regulated genes and 33 down-regulated genes in CRLM samples compared to primary CRC samples in the GSE81582 dataset (**Figure 1B**). Details of these two differential gene sets were recorded in (**Supplementary Tables 1**, **2**). Ninety-six genes were identified after extracting the intersection of the two differential gene sets (**Figure 1C**).

### Developing and evaluating of a prognostic model

**Figure 2:**
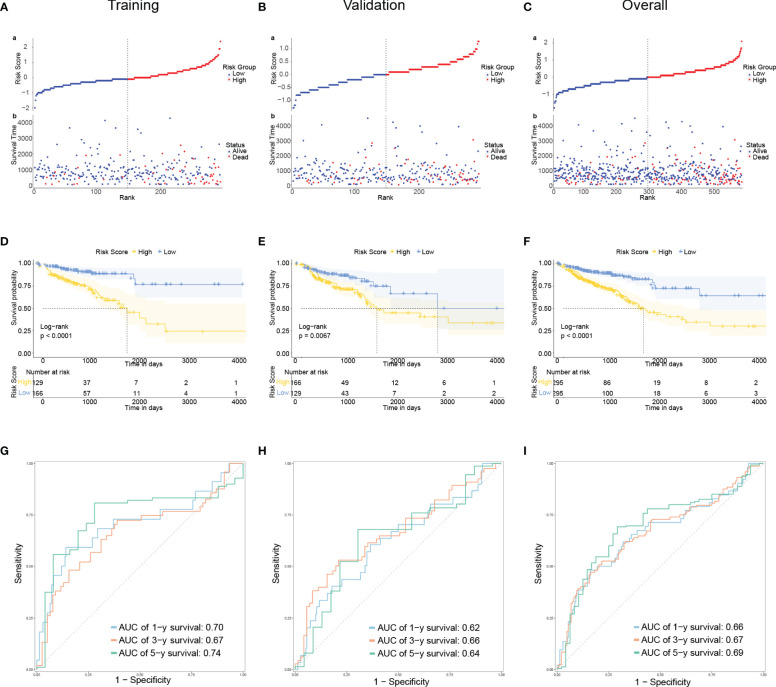
Developing and evaluating of a prognostic model **(A–C)**: The distribution of the risk scores, overall survival status, and survival time in the training **(A)**, validation **(B)**, and overall **(C)** groups. **(D–F)**: The Kaplan–Meier curves for survival status and survival time in the training **(D)**, validation **(E)**, and overall **(F)** groups. The x-axis represents the survival time, while the y-axis represents the survival probability. The blue line represents patients with low-risk scores, while the yellow line represents those with high-risk scores. **(G–I)**: Receiver operating characteristic (ROC) curves demonstrated the potential of the CRLM-associated signature in predicting 1-year, 3-year, and 5-year overall survival (OS) in training **(G)**, validation **(H)**, and overall **(I)** groups.

The TCGA sample data were randomly divided into training and validation groups at a 1:1 ratio (**Table 1**). The training and validation groups were used to study the prognostic importance of CRLM-related genes by constructing and validating CRLM-related prognostic features together with the overall group. Using univariate Cox analysis, we discovered CRLM genes that are related to prognosis. Then, using LASSO analysis, 7 important CRLM-related genes (HAMP, COLEC11, MMP3, UGT2B7, C8G, SERPINA1, IFITM10) were further identified, and signatures were constructed. (**Figure 1D, E**). The following formula was used to compute the risk score: r $isk\;score=\sum_{i=1}^{7}Coef_{i}*X_{i}$ . The $Coef_{i}$ for each gene and the expression of each gene in all samples can be found in **Supplementary Table 3**. Patients were categorized based on their median risk score. This sample distribution was reasonable in both three groups according to the distribution of risk scores and the distribution of survival status. (**Figures 2A–C**). According to Kaplan-Meier analysis, the high-risk group’s OS was significantly lower in the training, validation, and overall groups compared to the low-risk group (**Figures 2D–F**). We displayed time-dependent ROC curves to measure the prediction model’s effectiveness and calculated the area under the curve (AUC). The Decision Curve Analysis (DCA) plots effectively demonstrated the clinical utility and net benefit of our model across a range of threshold probabilities (**Supplementary Figure 1**). The results showed that this prediction model was valuable in the training, validation, and overall groups in predicting OS in the short and long term. (**Figures 2G–I**).

**Table 1:** The clinical characteristics of colorectal cancer patients in the training and validation group.

Characteristics		Training Group (N=295)		Validation Group (N=295)		P
		NO.	%	NO.	%	
Sex	Female	135	45.76%	134	45.42%	0.93
	Male	160	54.24%	161	54.58%	
Age	≤67 (Median)	147	49.83%	149	50.51%	0.87
	>67 (Median)	148	50.17%	146	49.49%	
Pathologic stage	I	50	16.95%	53	17.97%	1.00
	II	107	36.27%	106	35.93%	
	III	86	29.15%	84	28.47%	
	IV	43	14.58%	42	14.24%	
	Unknown	9	3.05%	10	3.39%	
T	T1	10	3.39%	11	3.73%	0.57
	T2	50	16.95%	53	17.97%	
	T3	208	70.51%	194	65.76%	
	T4	27	9.15%	36	12.20%	
	Unknown	0	0.00%	1	0.34%	
N	N0	171	57.97%	166	56.27%	0.29
	N1	77	26.10%	68	23.05%	
	N2	47	15.93%	61	20.68%	
M	M0	218	73.90%	221	74.92%	0.98
	M1	43	14.58%	41	13.90%	
	Mx	29	9.83%	29	9.83%	
	Unknown	5	1.69%	4	1.36%	
Survival Time	Long (>5 years)	25	8.47%	23	7.80%	0.76
	Short (<5 years)	270	91.53%	272	92.20%	
OS status	Dead	54	18.31%	68	23.05%	0.16
	Alive	241	81.69%	227	76.95%	

### Developing and evaluating of a nomogram

**Figure 3:**
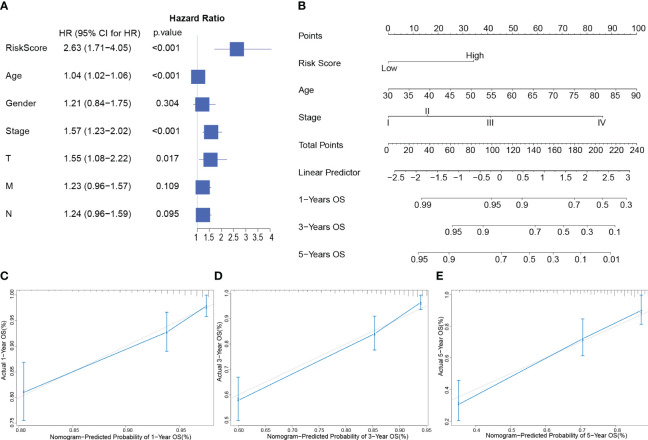
Developing and evaluating of a Nomogram **(A)**: Forest plot of multivariate Cox analysis of clinical factors and risk scores with OS. **(B)**: Nomogram for predicting the 1-, 3-, and 5-year OS of CRC patients. **(C–E)**: Calibration plots show the association of predicted 1- **(C)**, 3- **(D)**, and 5- **(E)** year OS with actual survival duration. The grey dotted line represents the ideal predictive model, while the solid blue line represents the actually predicted survival probability through the model.

Risk Score, Age, and AJCC Stage were independent indicators of CRC prognosis by multivariate Cox regression (**Figure 3A**). Based on the findings, a nomogram (**Figure 3B**) was developed to predict OS by combining risk score with age and AJCC stage. Finally, calibration curves were used to assess the nomogram’s prediction capabilities. According to the statistics, actual survival times were quite close to the predictions of this nomogram for 1, 3, and 5 years (**Figures 3C–E**). The information above indicates that this nomogram has a strong predictive ability.

### Relationship between immunotherapy and predictive model

**Figure 4:**
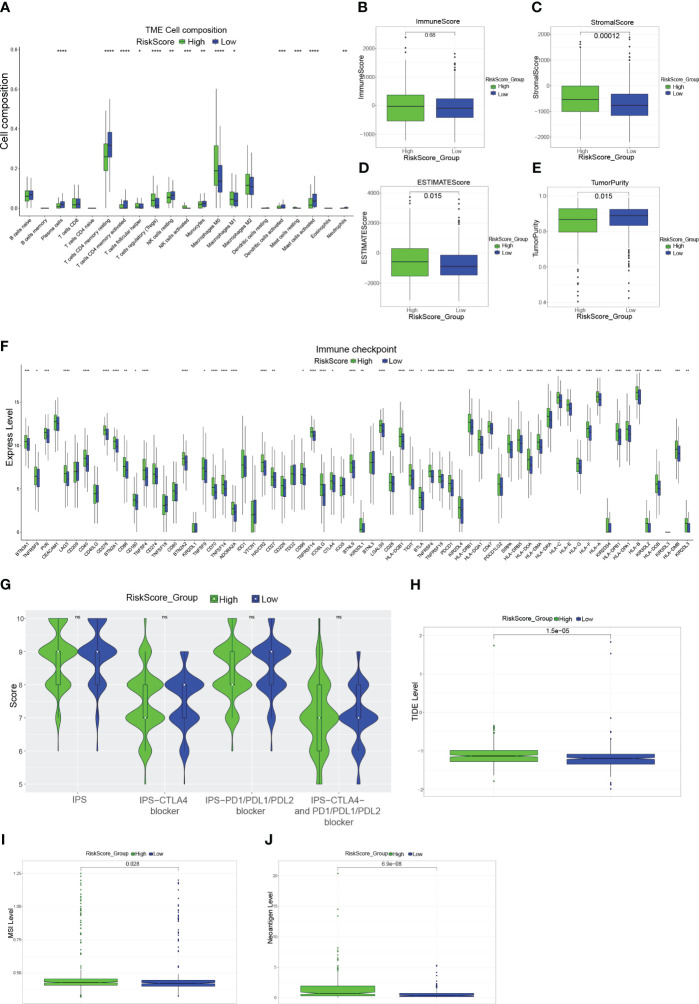
Immune-related analysis of CRC patients **(A)**: Box plot showing the proportion of immune cells in the different risk groups. **(B–E)**: Box plots evaluating differences in the immune score **(B)**, stromal score **(C)**, ESTIMATE score **(D)**, and tumor purity **(E)** in high and low-risk groups. **(F)**: Expression of immune checkpoint genes in the high-risk and low-risk groups. **(G)**: Violin plot showing the difference in values of IPS, IPS-CTLA4 blockers, IPS-PD1/PD-L1/PD-L2 blockers, and IPS-CTLA4 and PD1/PD-L1/PD-L2 blockers in the high-risk and low-risk groups. **(H, I)**: Box plots evaluating differences in TIDE score **(H)**, MSI level **(I)**, and Neoantigen level **(J)** in high and low-risk groups. *P<0.05, **P<0.01, ***P<0.001, ****P<0.0001.

First, comparing the percentage of immune infiltrating cells in the different risk groups, statistically, significant differences were found for Plasma cells, Some T cells (**Figure 4A**). Although there was no difference in the immune scores, in the high-risk group, the score of stromal and ESTIMATE was significantly higher (P< 0.05), and the tumor purity was significantly lower in the high-risk group (**Figures 4B–E**). The clinical use of ICIs was guided by comparing the differences in immune checkpoint gene expression levels between the high- and low-risk groups (**Figure 4F**), and many checkpoint genes differed significantly between the two groups, including the potent immunotherapeutic targets PDCD1 (PD-1), CD274 (PD-L1), and CTLA4. To assess the efficacy of immune checkpoint inhibitor treatment, the IPS was used (**Figure 4G**). There were no statistically significant differences in IPS, IPS-CTLA4 blockers, IPS-PD1/PD-L1/PD-L2 blockers, or IPS-CTLA4 and PD1/PD-L1/PD-L2 blockers between the two groups. Interestingly, the TIDE scores were significantly higher in the high-risk group than in the low-risk group (**Figure 4H**). Furthermore, in the high-risk group, the MSI and Neoantigen levels were significantly higher (**Figure 4I, J**).

### Sensitivity to anti-cancer drugs in various risk scores

**Figure 5:**
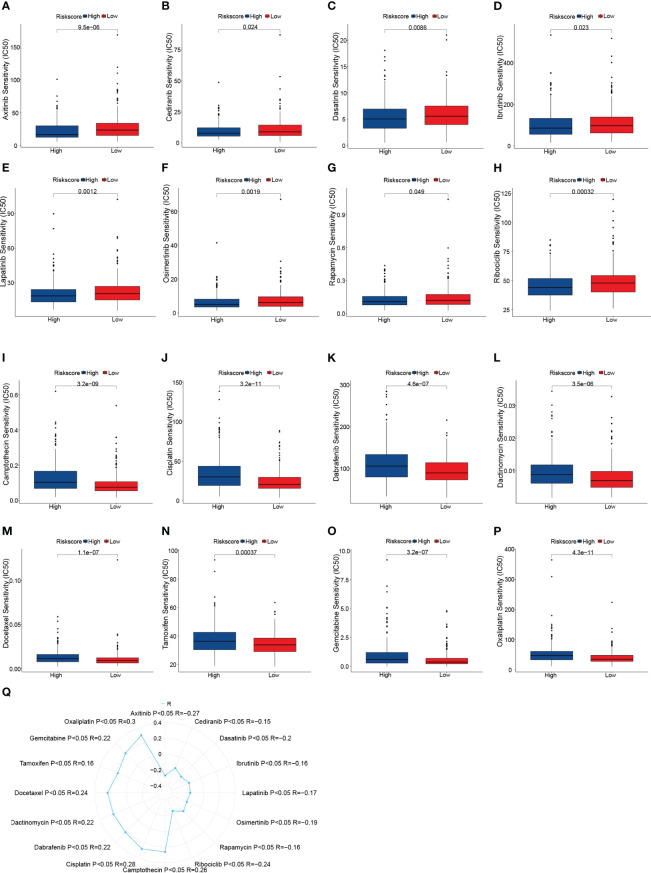
Drug sensitivity analysis. **(A–P)** Box plots of sensitivity analysis for Axitinib **(A)**, Cediranib **(B)**, Dasatinib **(C)**, Ibrutinib **(D)**, Lapatinib **(E)**, Osimertinib **(F)**, Rapamycin **(G)**, Ribociclib **(H)**, Camptothecin **(I)**, Cisplatin **(J)**, Dabrafenib **(K)**, Dactinomycin **(L)**, Docetaxel **(M)**, Tamoxifen **(N)** Gemcitabine **(O)**, and Oxaliplatin **(P)** in patients at low and high risk. **(Q)** Radar plot of correlation between drug sensitivity and Risk scores.

In CRC patients, we estimated the IC50 of 198 chemotherapy medications or inhibitors and determined the efficacy of the risk score as a predictor of chemotherapy response. The estimated IC50 for Axitinib, Cediranib, Dasatinib, Ibrutinib, Lapatinib, Osimertinib, Rapamycin, and Ribociclib in the high-risk group was significantly lower (**Figures 5A–H**). Additionally, compared to the high-risk group, the IC50 of Camptothecin, Cisplatin, Dabrafenib, Dactinomycin, Docetaxel, Tamoxifen, Gemcitabine, and Oxaliplatin was significantly lower in the low-risk group (**Figures 5I–P**). Finally, the correlation between the risk score and the above drugs was calculated using the Spearman rank correlation coefficient (**Figure 5Q**). These findings show that the risk score is connected to the drug’s sensitivity.

### HAMP highly expressed in both data sets

**Figure 6:**
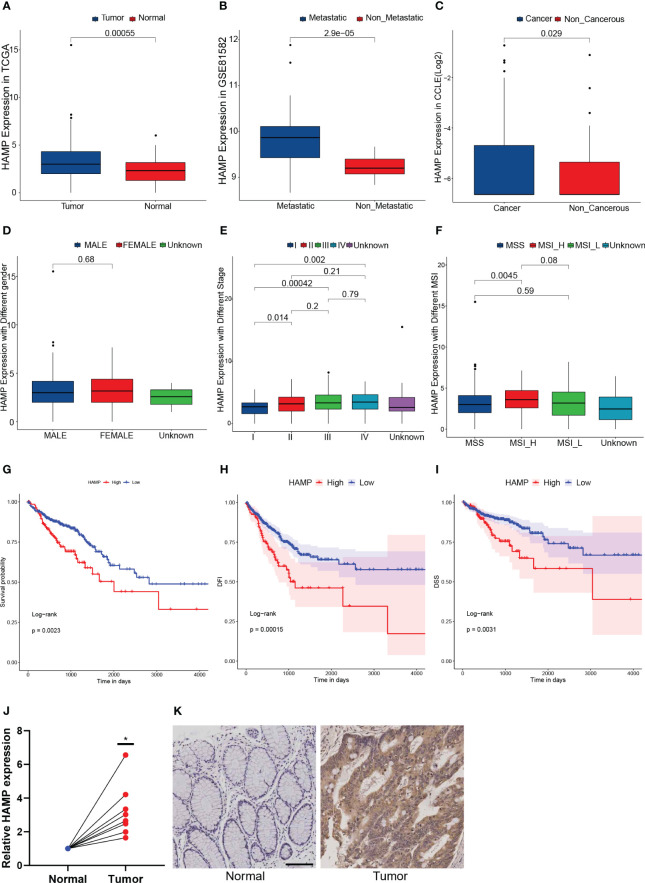
Hepcidin antimicrobial peptide (HAMP) highly expressed in both data sets **(A)**: The box plot illustrates the difference in HAMP expression between tumor and normal samples. **(B)**: The box plot illustrates the difference in HAMP expression between metastatic samples and non-metastatic CRC samples. **(C)**: The box plot illustrating the difference in HAMP expression in CRC and non-cancerous cell lines. **(D)**: The box plot illustrates the difference in HAMP expression between male and female samples. **(E)**: The box plot illustrating the difference of HAMP expression in samples with different stages. **(F)**: The box plot illustrating the difference of HAMP expression in samples with different MSI. **(G–I)**: The Kaplan-Meier survival curves show that the OS **(G)**, disease-specific survival (DSS) **(H)**, and disease-free interval (DFI) **(I)** of the High HAMP group were significantly lower than those of the Low HAMP group. **(J)**: The mRNA expression of HAMP in eight pairs of normal and tumor samples. (n = 8, *: p< 0.05) **(K)**: The immunohistochemistry (IHC) of HAMP in normal and tumor samples.

Of the 7 genes in this prognostic model, HAMP was the only gene that was highly expressed in both data sets of CRC and CRLM simultaneously (**Figure 6A, B**, **Supplementary Table 4**). To verify the correlation between HAMP expression and CRC, we further explored the expression of HAMP in a variety of CRC cell lines in the CCLE database. HAMP expression was higher in the CRC cell line than in the normal cell line (p = 0.029) (**Figure 6C**). However, between samples of different genders, there was no significant difference in HAMP expression (**Figure 6D**). The expression of HAMP in cancer samples ascended with the worsening of the stage (**Figure 6E**). Moreover, the expression of HAMP in stage I was significantly lower than that in stage II (p = 0.014), III (p = 0.00042), and IV (p = 0.002) (**Figure 6E**). There was no significant expression difference in different MSI (**Figure 6F**). When compared to the high MSI (MSI-H) group, HAMP expression was considerably lower in the microsatellite stability (MSS) group (p = 0.0045) (**Figure 6F**). Demonstrated that HAMP is related to CRC and the progress of cancers. In the TCGA dataset, we divided the samples into the High HAMP group and the Low HAMP group according to the median of HAMP expression. According to the survival study, the high HAMP group had significantly lower rates of OS (P = 0.0023), disease-free interval (DFI) (P = 0.00015), and disease-specific survival (DSS) (P = 0.0031) (**Figures 6G–I**). Indicated that HAMP could function as an independent biomarker for OS prediction. To further verify HAMP expression in the CRC samples, In eight pairs of clinical samples, we employed qRT-PCR and IHC to confirm our findings. The results revealed that HAMP expression was significantly higher in CRC samples than in control samples at both the mRNA (**Figure 6J**) and protein (**Figure 6K**) levels (p< 0.05).

### Analysis of HAMP in pan-cancer

**Figure 7:**
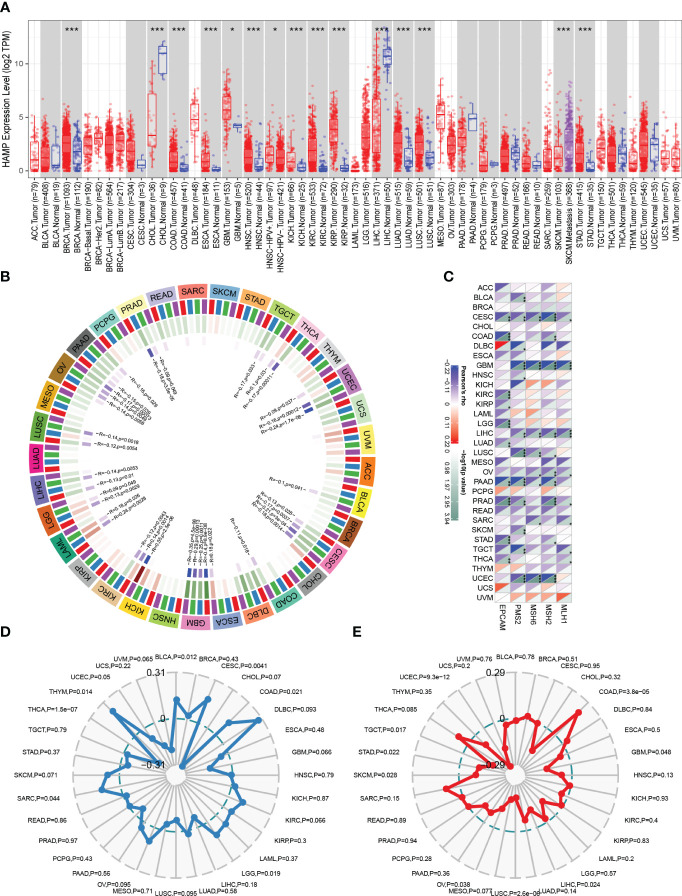
Analysis of HAMP in Pan-Cancer **(A)**: The box plot illustrating the difference in HAMP expression between tumor samples and normal samples in different cancer types in TIMER analysis. **(B)**: The circular graph shows the interaction of HAMP with the four major DNA methyltransferases in pan-cancer. The first outer ring is the cancer type, the second ring is the four DNA methyltransferases (DNMT1: red, DNMT2: blue, DNMT3A: green, DNMT3B: purple), the third ring is the correlation coefficient, and the fourth ring is the p-value. **(C)**: Heatmap shows how HAMP interacts with five DNA mismatch repair genes across various cancer types. The bottom right triangle is colored to represent p-values, while the top left triangle is colored to represent correlation coefficients for each association. **(D, E)**: The radar chart shows the association of HAMP with TMB **(D)** and MSI **(E)** in each cancer type.

HAMP expression was significantly increased in most cancer tissues compared to normal tissues, including BRCA, COAD, ESCA, GBM, HNSC, KICH, KIRC, KIRP, LUAD, LUSC, and STAD (**Figure 7A**). Tumors regularly have altered DNA methylation states, and the DNA methyltransferase family is primarily responsible for DNA methylation. In the majority of cancer types, HAMP was significantly correlated with the four main methyltransferases (**Figure 7B**). Furthermore, (**Figure 7C**) shows the correlation of HAMP with DNA mismatch repair genes in pan-cancer. TMB of various tumor samples was counted separately. In BLCA, CESC, COAD, LGG, SARC, and THYM, HAMP was associated with TMB positively, but in THCA, HAMP was associated with TMB negatively. (**Figure 7D**). Finally, we noted that HAMP was positively correlated with MSI in COAD, whereas it was negatively correlated with MSI in GBM, LIHC, LUSC, OV, SKCM, STAD, TGCT, and UCEC (**Figure 7E**).

### Identification of HAMP-related molecular functions and pathways

**Figure 8:**
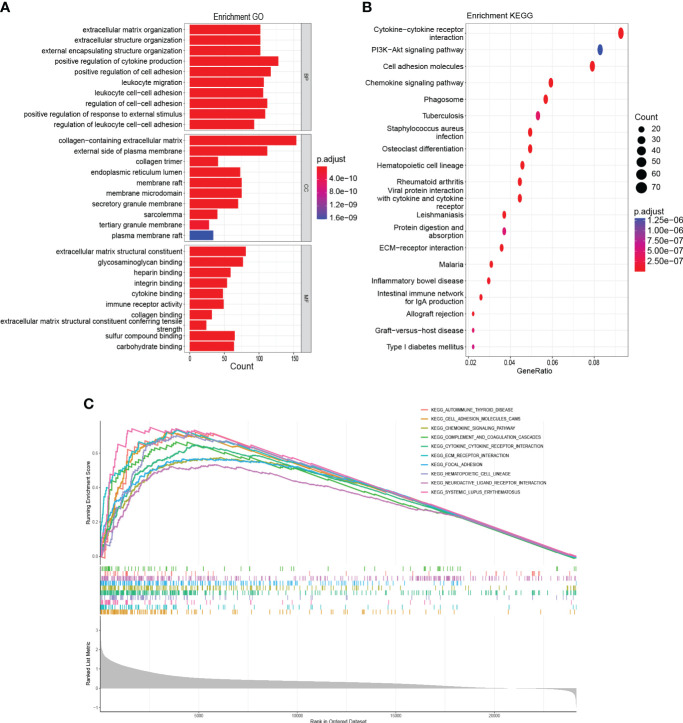
Functional enrichment analysis **(A)**: GO analysis on the biological processes (BP), cellular components (CC), and molecular functions (MF). **(B)**: KEGG enrichment pathway analysis. **(C)**: Activated pathways analyzed by gene set enrichment analysis (GSEA).

GO annotation findings indicated that HAMP-related genes were primarily involved in tumorigenesis and metastasis (e.g., positive regulation of cell activation, adhesion) and immune response-related processes (e.g., cytokine binding, immune receptor activity) (**Figure 8A**). KEGG analysis showed an association with tumorigenesis and metastasis pathways (e.g., PI3K-Akt signaling pathway, Cell adhesion molecules) and immune activation pathways (e.g., Cytokine-cytokine receptor interaction, Chemokine signaling pathway) (**Figure 8B**). Finally, GSEA was performed, and the same results showed that the tumorigenesis, metastasis pathways, and immune activation pathways were mainly involved (**Figure 8C**).

### Relationship between immunotherapy and HAMP

**Figure 9:**
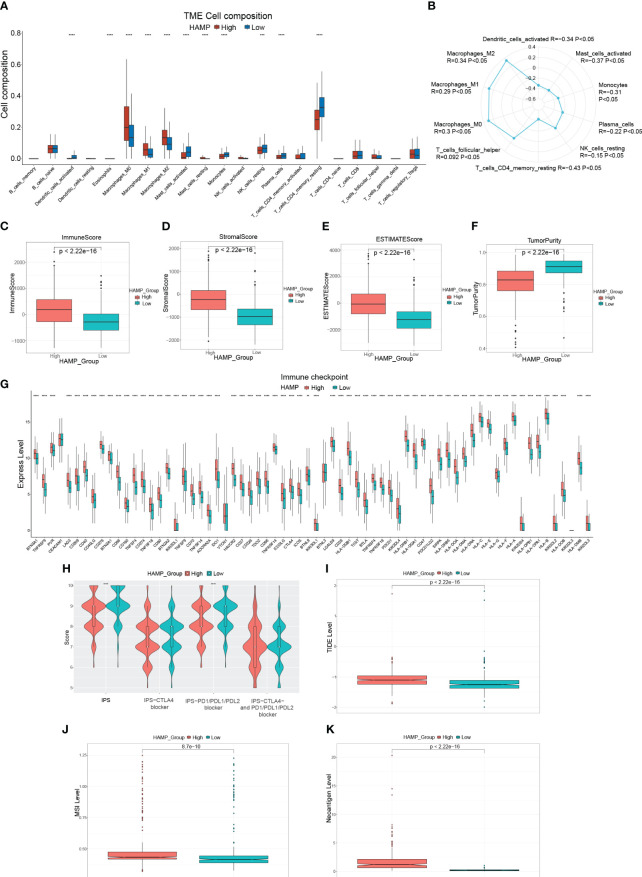
Immune-related analysis of HAMP in CRC patients **(A)**: Box plot showing the relative proportion of immune cells in the different HAMP expression groups. **(B)**: Radar chart illustrating the correlation between HAMP and different immune cells. **(C–F)**: Box plots evaluating differences in the immune score **(C)**, stromal score **(D)**, ESTIMATE score **(E)**, and tumor purity **(F)** in different HAMP expression groups. **(G)**: Box plots evaluating the expression of immune checkpoint genes in different HAMP expression groups. **(H)**: Violin plot showing the difference in values of IPS, IPS-CTLA4 blockers, IPS-PD1/PD-L1/PD-L2 blockers, and IPS-CTLA4 and PD1/PD-L1/PD-L2 blockers in different HAMP expression groups. **(I–K)**: Box plots evaluating differences in TIDE score **(I)**, MSI level **(J)**, and Neoantigen level **(K)** in different HAMP expression groups. *P<0.05, **,P<0.01, ***P<0.001, ****P<0.0001.

First, the percentage of immune infiltrating cells in the different HAMP expression groups was compared. HAMP expression was positively connected with macrophages (M0, M1, M2), whereas it was inversely related to dendritic cells activated, T cells CD4 memory resting, mast cells activated, plasma cells, and monocytes (**Figure 9A, B**, **Supplementary Figure 2**). The high expression group had substantially higher immune, stromal, and ESTIMATE scores (P< 0.05). In the meantime, the tumor purity in the high-expression group was significantly lower (P< 0.05) (**Figures9C–F**). Clinical use of ICIs was guided by comparing differences in immune checkpoint gene expression levels between the high- and low-expression groups (**Figure 9G**), and many checkpoint genes differed significantly between the two groups, including powerful targets PD-1/L1 and CTLA4. Response to immune checkpoint inhibitor therapy was assessed with the IPS Score (**Figure 9H**). The IPS, IPS-PD1/PD-L1/PD-L2 blockers scores were significantly lower in the high expression group. Additionally, the group with solid expressiveness had significantly higher TIDE scores (**Figure 9I**). The levels of MSI and Neoantigen were considerably more significant in the group with high expression (**Figure 9J, K**). These findings imply an essential link between HAMP expression and immunotherapy.

### Sensitivity to anti-cancer drugs in various HAMP expression

**Figure 10:**
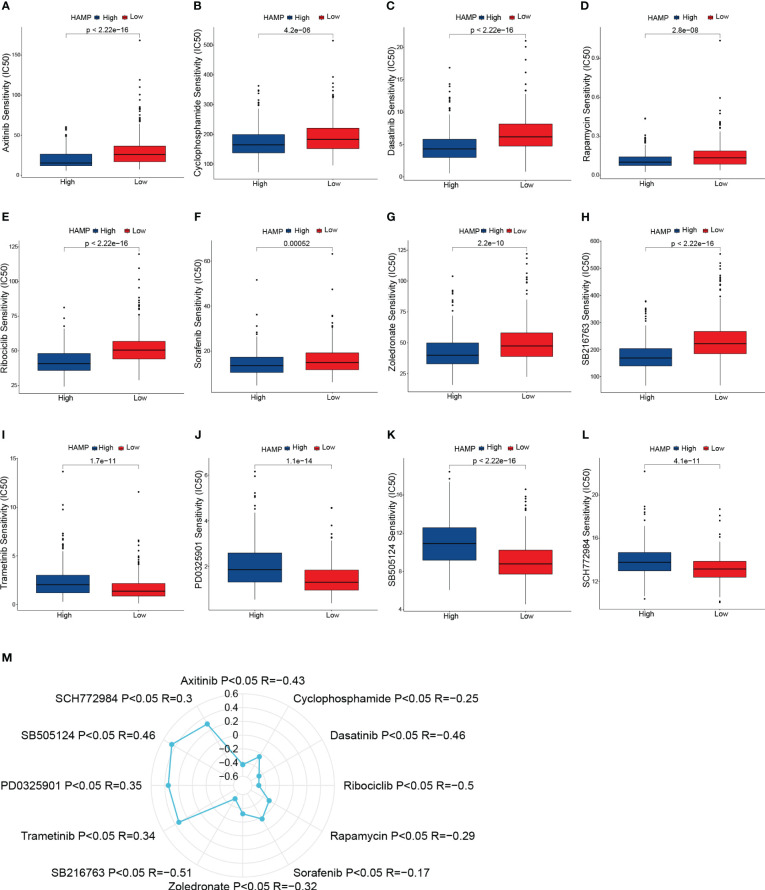
Drug sensitivity analysis. **(A–L)**: Box plots of sensitivity analysis for Axitinib **(A)**, Cyclophosphamide **(B)**, Dasatinib **(C)**, Rapamycin **(D)**, Ribociclib **(E)**, Sorafenib **(F)**, Zoledronate **(G)**, SB216763 **(H)**, Trametinib **(I)**, PD0325901 **(J)**, SB505124 **(K)** and SCH772984 **(L)** in patients at different HAMP expression group. **(M)**: Radar plot of correlation between drug sensitivity and HAMP expression.

We estimated the IC50 of 198 drugs or inhibitors in CRC patients to determine the validity of HAMP expression profile as a predictor of chemotherapy response. The estimated IC50 for Axitinib, Cyclophosphamide, Dasatinib, Rapamycin, Ribociclib, Sorafenib, Zoledronate, and SB216763 was noticeably lower in the high expression group (**Figures 10A–H**). Moreover, the estimated IC50 for Trametinib, PD0325901, SB505124, and SCH772984 in the low expression group was significantly lower compared to the high expression group (**Figures 10I–L**). Consequently, the connection of HAMP expression and the IC50 values of the above drugs was calculated using Spearman rank correlation coefficients (**Figure 10M**). These results reflect that the expression of HAMP is correlated with drug sensitivity.

### HAMP promotes the migration of CRC cells

**Figure 11:**
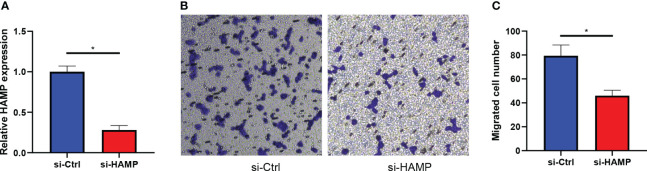
HAMP promotes the migration of CRC cells. **(A)**: Bar plots show the mRNA expression levels of HAMP in DLD-1 CRC cells in the control and si-HAMP groups (*: p< 0.05). **(B, C)**: The crystal violet staining of migrated cells by transwell migration assay. Transwell migration assays show the effects of inhibition of HAMP on the migration of DLD-1 CRC cells. Data represent the means ± SEM, * p< 0.05. n = 3 independent experiments.

We transfected the CRC cell line DLD-1 with si-RNA of HAMP (si-HAMP), and the qRT-PCR results showed that the expression of HAMP in the si-HAMP group was significantly lower than that in the control group (**Figure 11A**) in mRNA levels (p< 0.05). Then the transwell migration assay of the si-HAMP and control groups revealed that the number of migrated cells in the si-HAMP group was significantly lower than that in the control group (**Figure 11B**), indicating the silencing of HAMP in the CRC cells suppressed the cell migration. The statistical analysis of the transwell migration assay is shown in **Figure 11C** (p< 0.05).

## Discussion

The liver is the organ that CRC distant metastases most frequently affect (32). Nearly half of CRC patients develop liver metastases (33), and even after successful resection, most patients still experience disease recurrence (34). In order to identify potential targets and evaluate patient prognosis, critical genes associated with CRLM are essential. We analyzed mRNA expression profiles for CRC samples, CRLM, and primary CRC samples to screen for differentially expressed CRLM-associated genes. There were observed to be 96 differentially expressed genes in total. We then further created a new 7-genes prognostic model by LASSO regression and further calculated risk scores to classify CRC patients into high-risk and low-risk groups. The final performance of the model was validated in the training, validation, and overall groups, and the ROCs and DCAs verified its robustness in predicting 1-year, 3-year, and 5-year OS, demonstrating its reliable predictive ability for CRC patients’ prognosis. In multivariate Cox regression analysis, age, stage, and risk score could be used as independent indicators to assess the prognosis of CRC. Based on these findings, we developed a nomogram with strong predictive power.

Immunological characteristics of CRC influence immunotherapy response and patient clinical prognosis (35, 36). A significant portion of the TME is made up of immunological infiltrating cells, and the main component of anti-tumor immunity is the T-cell immune response (37), in addition to Plasma cells (38, 39), monocytes (40) and dendritic cells (41) play an anti-tumor immunity role, and Higher risk scores were shown to be adversely connected with the infiltration of T cells, plasma cells, monocytes and dendritic cells in our investigation. This suggests that a higher risk score may imply a poorer anti-tumor response. Significant components of the TME include immune and stromal cells, and high stromal and immune scores tend to have a poor prognosis (42, 43). As expected, in our study, the high-risk group had significantly higher immune scores, stromal scores, and ESTIMATE scores. Higher tumor purity in certain tumors predicts a relatively better prognosis (44, 45). Similarly, our study presented that the low-risk group had higher tumor purity. Although, in the high-risk group, the expression levels of numerous immunological checkpoint genes were much more significant, including much more potent immunotherapy targets PD-1/L1 and CTLA4, suggesting that blocking these immunological checkpoints may assist the high-risk population. We used the TIDE (46) score and IPS (47) to infer sensitivity to immunotherapy. But we found that, in the high-risk group, the TIDE score was significantly higher, and there was no noticeable difference in IPS. It indicates that the likelihood of immune evasion is high in the high-risk group, and ICI medication is nearly impossible to benefit patients. Overall, the high-risk group presented a negative immunotherapy result and prognosis. For tailored treatment to enhance patient prognosis, our subsequent investigation revealed chemotherapeutic drugs or inhibitors that are sensitive to various risk groups.

As the only differential gene overexpressed in both tumor and metastatic samples in the 7-genes signature associated with CRLM described above, we hypothesized that overexpression of HAMP may promote the development of CRC. Previous studies demonstrate that aberrant expression of HAMP could contribute to the progression of hepatocellular carcinoma by reducing the infiltration of cytotoxic immune cells because it failed to meet the iron requirement of immune cells (48). Iron acts as an oncogenic or co-carcinogenic agent that can induce hepatocellular carcinoma (49), and abnormal iron intake is an important risk factor for the development of hepatocellular carcinoma (50). In addition, increased levels of HAMP expression are positively correlated with CRC staging, depending on the status of tumor suppressors in CRC (51). Similarly, qRT-PCR showed significantly elevated HAMP mRNA compared to normal cell CRC cell lines, and IHC also showed higher HAMP protein levels in tumor tissues. The transwell assay showed that HAMP overexpression promoted tumor cell migration. Survival analysis showed that samples from the high HAMP group had significantly lower OS, DSS, and DFI than those from the low HAMP group. The above analysis suggests that HAMP overexpression may promote tumor development and is a potential prognostic biomarker that can distinguish CRC patients with different prognoses.

Analysis targeting the oncogenic role of HAMP and the associated immunological profile in pan-cancer showed that HAMP expression was significantly up-regulated in the majority of tumor types. HAMP overexpression was shown to be related to DNA methyltransferase. The presence of dMMR-MSI-H in some solid tumors is a clear biomarker of potential response to immunotherapy (52, 53). TMB is a valid biomarker for immune checkpoint inhibitor selection in certain cancer types (54). Our results showed that upregulation of HAMP is associated with DNA mismatch repair genes, TMB, and MSI with different cancers, demonstrating the crucial role of HAMP in predicting the effects of immunotherapy.

It is shown that HAMP is closely associated with immune regulation (55), and our enrichment analysis results suggested that HAMP-related genes are mostly engaged in immune activation pathways (cytokine-cytokine receptor interactions, chemokine signaling pathways) in CRC. However, in CRC, the relevance of HAMP to immune cell infiltration and stromal cells in the TME is still unclear. In our study, Plasma cell, monocyte, and dendritic cell infiltration were significantly higher in the low HAMP-expressing group. Similarly, the scores of IPS, IPS-PD 1/L1/L2 blockers were found to be higher in the low-expressing group, and the TIDE score was significantly lower in the low-expressing group than in the high-expressing group. The above results suggest that the low HAMP expression group may be more suitable for immunotherapy.

Our study has certain limitations because the data we used came from open databases, the results are retrospectively constructed and validated, and the value of the prognostic model needs to be validated in a larger clinical cohort. In addition, the mechanism of HAMP in promoting liver metastasis in CRC needs to be validated by further experiments.

## Conclusion

Overall, we constructed a prognostic model consisting of 7 CRLM-associated genes. As independent risk factors, age and AJCC Stage with risk scores were constructed nomogram. In addition, the derived gene HAMP helps to guide the exploration of profitable immunotherapeutic strategies.

## Data availability statement

The original contributions presented in the study are included in the article/**Supplementary Material.** Further inquiries can be directed to the corresponding authors.

## Ethics statement

The studies involving human participants were reviewed and approved by Medical Ethics Committee of the First Affiliated Hospital of Xi’an Jiaotong University. The patients/participants provided their written informed consent to participate in this study.

## Author contributions

All authors participated in the conception and design of the study. CL, ZL, JY and FL performed the bioinformatics analysis and visualization. DX, XH, WZ prepared the Table and Figure. CL, QS, FL wrote the manuscript, and WH and WZ revised the manuscript. All authors have read the manuscript and approved the final version. All authors contributed to the article and approved the submitted version.
