# Supplementary material for: Construction of a prognostic signature associated with liver metastases for prognosis and immune response prediction in colorectal cancer
Source: Front Oncol. 2023 Jul 26;13:1234045. doi: 10.3389/fonc.2023.1234045 (PMC10411999; doi:10.3389/fonc.2023.1234045)
Supplement: Supplementary file 1 [file DataSheet_1.docx]

Supplementary Material

Construction of a prognostic signature associated with liver metastases for prognosis and immune response prediction in colorectal cancer

Chang Liu ^1, †^, Zhihua Lu ^2, †^, Jun Yan ^1^, Dong Xue ^1^, Xiaoyu He ^3^, Wenbo Huang ^1^, Qi Sun ^1^, Wei Zhao ^1, *^ and Fanni Li ^4, *^

^1^ Department of General Surgery, The First Affiliated Hospital of Xi'an Jiaotong University, Xi'an, China

^2^ Department of General Surgery, Qilu Hospital (Qingdao), Cheeloo College of Medicine, Shandong University, Qingdao, China

^3^ Center for Gut Microbiome Research, Med-X Institute, The First Affiliated Hospital of Xi'an Jiaotong University, Xi’an, China

^4^ Department of Talent Highland, The First Affiliated Hospital of Xi’an Jiaotong University, Xi’an, China

^†^ These authors contributed equally to this work.

*** Correspondence:**Corresponding Author:
Wei Zhao
zhaowei9803@126.com
Fanni Li
fannycpu@163.com

## Supplementary Figures


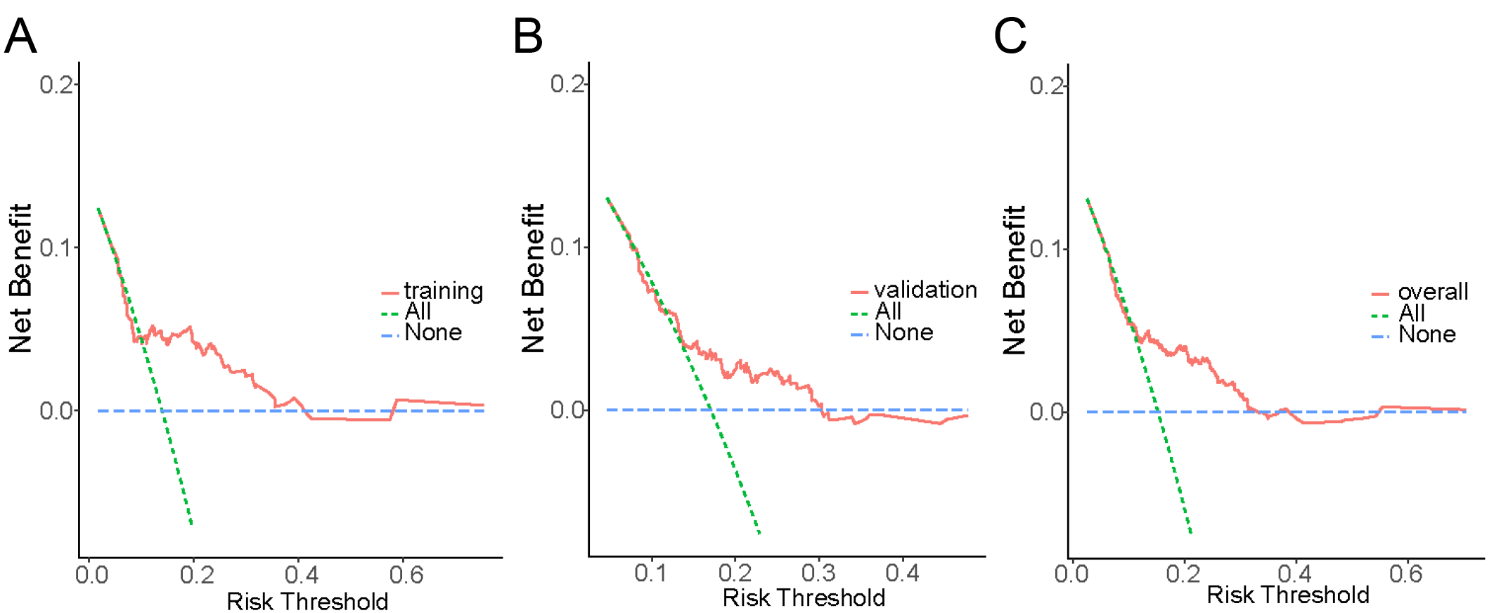


**Supplementary Figure 1.** The Decision curve analysis (DCA) plots show the clinical utility and net benefit of CRLM-related features in predicting the training group (A), validation group (B), and overall group (C) at various threshold probabilities. The x-axis represents the threshold probability and the y-axis represents the net benefit.


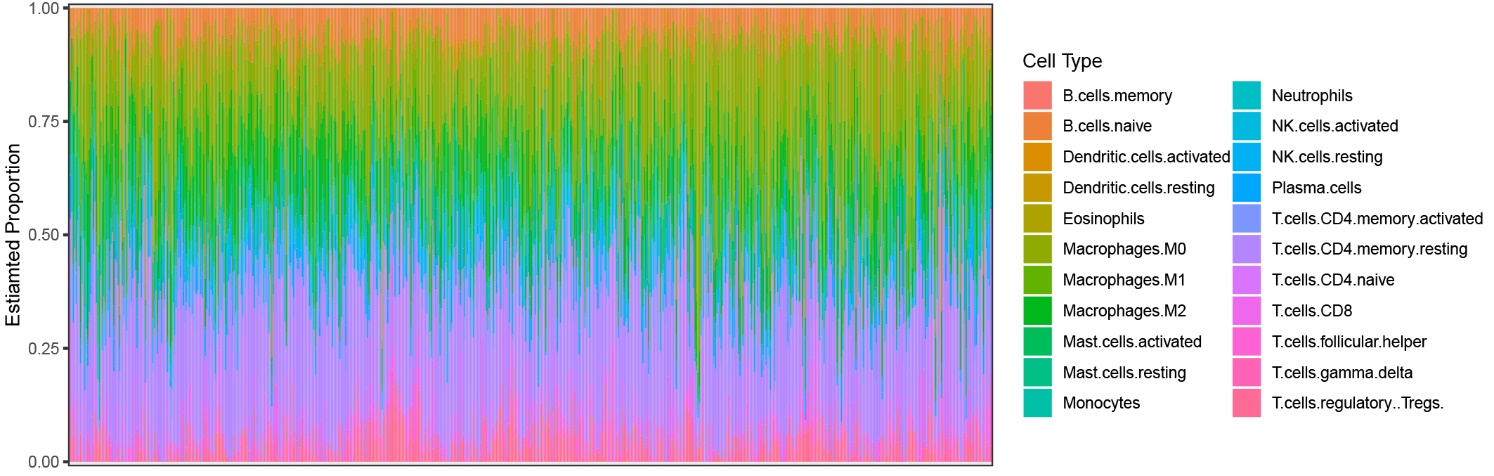


**Supplementary Figure 2.** The bar graphs exhibiting the proportion of tumor-infiltrating immune cells between the different HAMP expression groups.
